# Implementation of circulating cell-free DNA screening for fetal aneuploidies

**DOI:** 10.1515/almed-2025-0055

**Published:** 2025-03-25

**Authors:** Irene Madrigal Bajo, Meritxell Jodar Bifet, Celia Badenas Orquin

**Affiliations:** Service of Biochemistry and Genetics, 16493 Clinical Hospital of Barcelona and FCRB-Institute of Biomedical Research August Pi I Sunyer (IDIBAPS), Barcelona, Spain; Rare Diseases Networking Biomedical Research Centre (CIBERER), Barcelona, Spain; Service of Biochemistry and Genetics, Clinical Hospital of Barcelona and FCRB-Institute of Biomedical Research August Pi I Sunyer (IDIBAPS), Unit of Genetics, Universitat de Barcelona, Barcelona, Spain; Service of Biochemistry and Genetics, Clinical Hospital of Barcelona and FCRB-Institute of Biomedical Research August Pi I Sunyer (IDIBAPS), Barcelona, Spain

**Keywords:** circulating cell-free DNA, prenatal screening, non-invasive prenatal test, prenatal diagnosis

## Abstract

**Introduction:**

Circulating cell-free DNA (cfDNA) consists of extracellular DNA fragments that circulate in the bloodstream and derived from apoptotic cells such as hematopoietic cells or placental trophoblast cells during pregnancy.

**Contents:**

cfDNA screening has been included in prenatal screening programs for the detection of chromosomal abnormalities. Unlike other invasive techniques, such as amniocentesis or chorionic villus sampling, cfDNA screening only requires a maternal plasma test. The use of advanced technologies for cfDNA testing, including DNA sequencing and SNP arrays, enables the detection of pregnancies at risk for trisomy 21, 18 or 13.

**Summary:**

This test has demonstrated a high accuracy and reliability, with detection rates exceeding 99 % for trisomy 21, and a very low rate of false-positive and false-negative results. In some countries, cfDNA screening has already been integrated in combined or universal prenatal screening programs.

**Outlook:**

As new technologies emerge and become widely available, more accurate prenatal tests will be developed for other genetic abnormalities.

## Introduction

Combined first-trimester (CFTS) and second-trimester (CSTS) screening have been used since the 1980s to detect the most common fetal aneuploidies, including trisomy of chromosomes 21 (T21), 18 (T18) and 13 (T13). This screening test has significantly evolved over the years. Currently, CFTS includes maternal blood tests for biochemical markers such as pregnancy-associated plasma protein (PAPP-A) and the free β-human chorionic gonadotropin (β-hCG). Second-trimester screening includes tests for alpha-fetoprotein (AFP), unconjugated estriol (uE3), β-hCG and inhibin A concentrations. In both cases, the nuchal translucency test is included. The accuracy and diagnostic performance of CFTS and CSTS have improved substantially to reduce false positive (FP) rates and minimize the need for confirmatory invasive procedures. In the 1990s, the detection rate (DR) of CFTS was around 90 %, with a FP rate of 5 %. When CFTS or CSTS indicate a high risk of aneuploidy, an invasive test (chorionic sampling or amniocentesis) is required, which is associated with a small risk of miscarriage. In 1997, Lo et al. discovered fetal DNA in maternal plasma [1]. This finding opened the avenue for the development of non-invasive prenatal tests based on the analysis of circulating cell-free DNA (cfDNA). In the last decade, multiple studies have demonstrated the efficacy of fetal maternal plasma cfDNA testing, also known as non-invasive prenatal screening (NIPS), to assess the risk for T13, T18 and T21. NIPS has been proven to have a higher sensitivity and specificity and a lower FP rate, as compared to combined screening. This evidence suggests that cfDNA testing could be used in combination with CFTS or for universal screening to improve the accuracy of genetic screening for fetal genetic abnormalities early in pregnancy. cfDNA has different origins and consists of extracellular DNA fragments that circulate freely in the bloodstream. All individuals have cfDNA in plasma, as this DNA is released during apoptosis of different types of cells, primarily hematopoietic cells [2]. In the recent years, apoptotic tumor cells have been found to release a large amount of DNA into the bloodstream. Hence, it is used as a non-invasive test in the follow-up of cancer [3], 4]. Likewise, testing of DNA released into the blood by transplanted organ cells is used for the monitoring and diagnosis of transplant rejection [5], 6]. The plasma of pregnant women contains maternal and fetal cfDNA, with the latter being released by apoptotic trophoblasts [7], 8]. Fetal cfDNA can be detected from the 6th week of pregnancy and the whole fetal DNA is represented in it. Since cfDNA derives from the placenta, it disappears after birth. Fetal cfDNA is 150–200 pb in length, slightly shorter than maternal cfDNA.

This difference makes it possible to distinguish maternal from fetal cfDNA fragment, in some of the methodologies used, and to quantify the fetal fraction, i.e. the percentage of cfDNA that is of fetal origin.

## Prenatal NIPS methods

The last decade has witnessed significant advances in NIPS technologies, which currently enable screening for multiple genetic abnormalities in an accurate and non-invasive way for the fetus (Table 1).

**Table 1:** Most relevant capabilities of cfDNA screening technologies.

Characteristics	NGS	SNPs	RCR
Minimum gestational age	10	9	10
Detects common trisomies	YES	YES	YES
Detects rare trisomies	YES	YES	NO
Detects sex chromosome aneuploidies	YES	YES	YES
Detects triploidies	NO	YES	NO
Detects UPD	NO	YES	NO
Detects CNVs	>7 Mb	YES^c^	NO
Detects mosaicisms^a^	YES^b^	NO	NO
Twin pregnancy	YES	YES	YES
Quantifies FF	YES	YES	NO
Determines zygosity	NO	YES	NO

Modified from Benn et al., 2023. ^a^Description in page 9. ^b^YES, if the percentage of mosaicism is high. ^c^Depending on the array design. UPD, uniparental disomy; CNV, copy number variants; NGS, next generation sequencing; SNP, single nucleotide polymorphisms; RCR, rolling circle replication; FF, fetal fraction.

### Massive parallel sequencing

Massive parallel sequencing is one of the most widely-used techniques for studying cfDNA. This technology yields millions of sequence reads that are aligned to a reference human genome. The number of reads is counted to assess a potential excess (trisomy) or deficit (monosomy) of these sequences, which indicates the presence of an aneuploidy in a particular chromosome. In addition, by massive parallel sequencing, the study can be extended to the rest of chromosomes to identify copy number variants (CNV) exceeding 7 Mb.

### Single-nucleotide polymorphisms (SNP) testing

Arrays for the detection of SNP polymorphisms estimate the relative proportion of maternal and fetal genotypes to differentiate the two origins [9], 10]. This technique enables estimating the maximum probability that the fetus has or not an aneuploidy. Unlike massive parallel sequencing, SNP arrays require the analysis of plasma (maternal and fetal cfDNA) and maternal cellular DNA. This method detects triploidies and uniparental disomies (UPD) [11], 12].

### Direct cfDNA quantification or rolling circle replication (RCR)

RCR converts chromosomal fragments into quantifiable digital objects. This technique does not require PCR amplification or sequencing [13], 14], thereby being more cost-effective and affordable. RCR has a high sensitivity and specificity for the detection of aneuploidies, even in the presence of a low fetal fraction, which improves its robustness and clinical utility [15], 16]. However, only four different fluorophores (colors) can be currently used, which limits detection to only the most common aneuploidies (T21, T18 and T13) and sex chromosome aneuploidies (SCA).

## Sensitivity and specificity for the detection of chromosomal aneuploidies

### Aneuploidies of chromosomes 21, 18 and 13

Maternal plasma cfDNA screening is a cutting-edge technology that enables non-invasive screening for trisomies T21, T18 and T13. This test can be performed from the 10th week of pregnancy, since at this point, the percentage of fetal cfDNA can exceed 3 %, thereby enabling enhanced pregnancy monitoring. Two large studies have reported a detection rate of T21, T18 and T13 for the pregnant population with a high CFTS (≥1:200) of ∼2.3 % for T21; 0.4 % for T18; and 0.4 % for T13 [17], [18], [19]. In turn, in non-selected pregnant women, detection rates are 0.3–0.5 % for T21; 0.07–0.1 % for T18 and 0.05–0.08 % for T13 [19], 20]. The sensitivity and specificity allow assessment of the accuracy and effectiveness for cfDNA testing in the context of prenatal screening. There is a variety of NIPS methods available. However, these methods have a similar diagnostic performance for T21, T18 and T13, especially in singleton pregnancies. Most validation studies assessed the diagnostic performance of NIPS for aneuploidies using maternal plasma samples from women who underwent an invasive diagnostic test [detection of false negative (FN) results] or in affected and non-affected live-born neonates [detection of false negative or false positive (FP) results]. Sensitivity indicates the performance of the test in detecting fetus with specific aneuploidies. For T21, sensitivity is generally very high, close to 99.7 % (95 % CI, 99.1–99.9 %). Sensitivity is slightly lower for T18, reaching 97.8 % (95 % CI 94.9–99.1 %). Finally, the sensitivity for T13 is 97.0 % (95 % CI 65.8–100 %) [19], [21], [22], [23], [24]. The combined FP rate is 0.13 %, exceeding that for T18 and T13. In turn, specificity explains the test's performance in identifying fetuses without aneuploidies; for example, when cfDNA estimates a low-risk result, the fetus does not actually have an aneuploidy. In case of an aneuploidy, the result would be a FN. The reported specificity for common aneuploidies exceeds 99 % [19], [21], [22], [23], [24]. Its high sensitivity and specificity render NIPS a reliable and useful tool for early detection of genetic abnormalities such as T21, T18 and T13.

Positive predictive values (PPV) and negative predictive values (NPV) are essential when assessing the accuracy and clinical utility of NIPS. These values are influenced by several factors, including the prevalence of the condition, the fetal cfDNA fraction, and the sensitivity and specificity of the test used. PPV and NPV provide information about the probability that a positive or negative result is correct. PPV indicates the probability that a fetus has an aneuploidy when the test indicates a high risk. PPV is calculated by dividing the number of true positive results by the number of true positive results plus the number of FP. NPV indicates the probability that a fetus does not have an aneuploidy when the result indicates a low risk. NPV is calculated by dividing the number of true negative results by the number of true negative results plus the number of FN results. Techniques with high sensitivity (ability to detect true positive results) and high specificity (ability to detect true negative results) improves both predictive values. For instance, the PPV may be lower for T18 than for T21 due to the lower sensitivity and prevalence of T18.

### Sex chromosome aneuploidies

SCAs encompass conditions such as Turner syndrome (45,X), Klinefelter syndrome (47,XXY), Triple-X syndrome (47,XXX) and other X- and Y-chromosome related disorders. The diagnostic performance in detecting SCAs is currently high [25], 26]. However, the performance is slightly lower for X monosomy, primarily due to higher rates of maternal and fetal mosaicism in this aneuploidy [27], 28]. Mosaicism is defined as the presence of genetically different cell populations in the same individual. The inclusion of SCAs in NIPS has been a topic of debate among clinicians. Some SCAs, such as Turner syndrome (45,X) or Klinefelter syndrome (47,XXY), may significantly affect health or fertility, respectively. However, their inclusion in NIPS is controversial. Some authors argue that these aneuploidies cause mild or no symptoms at all [29], and their introduction as an alternative screening test to combined screening may increase screening costs [20], 30]. This variability complicates decision-making for both obstetricians and parents.

### Rare autosomal aneuploidies

The detection of rare autosomal aneuploidies (RAA), i.e. autosomal chromosomes other than chromosomes 13, 18 and 21, is a useful chromosome screening tool. Although the presence of a complete trisomy is incompatible with life, mosaic complete trisomy – i.e. when trisomy is only present in a percentage of cells-, can be associated with an abnormal functioning of the placenta. For example, placental mosaic T16 is associated with problems in fetal growth. These pregnant women have a higher risk of preeclampsia and require a more specific follow-up during pregnancy. In the most severe cases, labor induction may be necessary. Various studies have included RAA testing in prenatal screening. The detection rate (DR) for RAA in the general pregnant population ranges from 0.18 to 0.32 %, with a PPV of 6 %. The most common RAA detected include T7, T8, T16, T20 and T22 [20], 31].

## Twin pregnancies

Detecting aneuploidies is more challenging in twin pregnancies than in singleton pregnancies. However, the sensitivity and specificity of NIPS in twin pregnancies is comparable to that in singleton pregnancies [32], 33]. It should be taken into account that, in twin pregnancies, cfDNA is released by the two fetuses, which may hinder the interpretation of the results. In monozygotic twins (20–30 % of multiple pregnancies), the two fetuses share the same DNA; therefore, they will be affected by the same aneuploidy. In dizygotic twins (60–70 % of multiple pregnancies), differentiating the contributions of each fetus is essential for detecting specific aneuploidies in each fetus. Differences in the contribution of each fetus to the total cfDNA may hinder the detection of specific aneuploidies in one of the fetuses. Only some of the techniques available detect the FF of each fetus. Consequently, it is mostly not possible to determine which fetus has a high risk for the detected aneuploidy, and the fetal fraction corresponding to each fetus. It is worth noting that the presence of discordant fetuses or vanishing twins may alter NIPS results [34]. The incidence of vanishing twins is 50 % for pregnancies with three or more gestational sacs; 36 % for twin pregnancies; and 20–30 % for pregnancies achieved by assisted reproduction [35].

## No-call rate

Failure of NIPS to return a result (no-calls) varies widely across studies, generally ranging from 1 to 5 % [12], 36]. In 50–75 % of cases, a result is obtained from repeat screening using a new blood sample. A low FF is one of the main causes of no-calls. Although solid evidence is not available, a low FF may be due to several factors, including a small placenta [37], 38], or a high maternal body mass index (BMI) causing more inflammation and necrotic adipose tissue in this pregnant population [39], [40], [41]. In 2018, Chan et al. performed a retrospective study demonstrating that pregnant women with “no-calls” in NIPS were at a higher risk of presenting chromosomal aneuploidies (6.5 vs. 0.2 %), preeclampsia (11 vs. 1.5 %) and gestational diabetes (23 vs. 7.5 %), as compared to controls [42]. Some studies suggest an association between no-calls in NIPS and the presence of specific chromosomal aneuploidies such as Turner syndrome or a higher risk of T13 or T18 [36], 43]. Autoimmune diseases of the mother have also been associated with no-calls, mainly due to inflammatory reactions that increase the percentage of maternal cfDNA or mask the presence of fetal DNA in maternal blood [44], 45].

## Factors that may affect the sensitivity and specificity of NIPS

Other factors may also influence NIPS results, and discordant results may be obtained. Several studies have reported that the main causes of discordant results include fetal or maternal mosaicism, the presence of vanishing twins or maternal tumors, among others [46], 47].

### Presence of fetal mosaicisms

The detection of mosaicism is one of the reported limitations of NIPS. The presence of placental or fetal mosaicisms may lead to FP or FN results [45]. Genetic information from placental tissues is known to be representative of the fetus. However, in most cases, mosaicism – mainly placental-is observed in 1–2% of karyotypes [27]. Whereas fetal cfDNA has a placental origin, DNA from amniotic fluid has a fetal origin. Therefore, in pregnancies with a diagnosis of confined placental mosaicism, NIPS and amniotic fluid results will be discordant. In pregnancies with confined placental mosaicism, NIPS yield a FP result, as aneuploidy is only present in a percentage of placental cells. In fetal mosaicism, NIPS yield a FN result, as the aneuploidy is only present in a percentage of fetal cells. The last possibility is generalized mosaicism, where mosaicism is present both, in the placenta and the fetus. NIPS results will be consistent with that of the fetus, provided that the two tissues have a similar percentage of cells containing the aneuploidy; otherwise said, that they have a similar mosaicism ratio. If the tissues have very different mosaicism ratios, the results will probably be discordant, being FP or FN. A recent study assessed the impact of mosaicism ratio on the PPV of NIPS [48]. The study revealed that the T13 mosaicism was the most common, followed by T18 and T21 mosaicism. In addition, significant differences were observed in the PPVs for the three trisomies between samples with a low mosaicism ratio vs. those with a high mosaicism ratio.

### Presence of maternal mosaicisms

Maternal mosaicism is defined as the presence of two or more populations of maternal cells with different genetic composition. The presence of maternal mosaicism can lead to discordant results. If a fraction of maternal cells has a chromosomal abnormality, it could be detected and interpreted as the fetus having a chromosomal abnormality. An example is the progressive preferential loss of the inactivated X chromosome in pregnant women of advanced maternal age, which results in a mosaic X monosomy in maternal cfDNA [49], 50].

### Vanishing twins

Vanishing twins are pregnancy losses occurring in the first gestational trimester involving the spontaneous reduction of one or more fetuses (in the case of multiple pregnancies). An important cause of fetal loss in multiple pregnancies is the presence of a chromosomal aneuploidy. In dizygotic twin pregnancies, each fetus may contribute a different FF, and the presence of a vanishing twin with a high fetal fraction that is discordant with the surviving fetus may yield misleading NIPS results [34], 46], 51], 52].

### Pregnancies resulting from *in vitro* fertilization (IVF)

In pregnancies resulting from IVF, the placenta is generally smaller than in spontaneous conception. As a result, FFs can be lower [37], 38]. However, some studies have excluded significant differences in FF between populations of pregnant women who underwent IVF vs. spontaneous conception [53], [54], [55].

### Maternal neoplasms

The presence of a malignant neoplasm is relatively rare in the pregnant population. However, this may occur in 1 in 1,000 cases, being breast cancer, melanoma and cervical cancer the most common types of cancer during pregnancy [56], 57]. As aforementioned, cfDNA can originate from apoptotic tumor cells, and the presence of maternal neoplasms can generate discordant or uninformative results in NIPS [58]. The incidental detection of maternal neoplasms in NIPS has been reported in several studies [59]. Indeed, the detection of multiple aneuploidies in NIPS may indicate the presence of maternal tumors. In these cases, the pregnant patient must be informed and referral to the Service of Oncology should be considered.

### Organ transplant and maternal blood transfusions

Another factor that may influence NIPS results is organ or bone marrow transplants, which may interfere with the interpretation of results [60]. An example is when the pregnant patient receives a transplant from a male donor. In pregnancies of a female fetus, NIPS may identify Y-chromosome sequences from the transplanted organ. Pregnant women receiving a bone marrow or organ transplant from a male donor should be informed that incorrect results may be obtained. In these cases, invasive screening for aneuploidies should be offered. On another note, if the pregnant woman has received a blood transfusion from a male donor, it is recommended to wait at least four weeks to perform NIPS [61].

### Autoimmune diseases

Several studies reveal that the FF may be altered by different inflammatory reactions [45], 47], 62]. For example, there are reports of thrombocytopenic purpura with a low FF [44], 45]. Inflammatory reactions may increase maternal cfDNA, and the proportion of fetal cfDNA decreases as maternal cfDNA increases.

## Pre-test and post-test genetic counseling

Informing the patient before and after genetic testing is crucial. In this case, the pregnant women should be aware of the advantages and disadvantages of each of the screening tests or the invasive testing. Genetic counselors should receive appropriate training on how to clearly explain the advantages, limitations and results of NIPS to future mothers. In some countries such as Spain, based on the risk of aneuploidy estimated by CFTS, the patient is given the option to choose between invasive testing or NIPS. In the pre-screening counseling visit, the patient must be informed about the results that can be obtained, along with the advantages and limitations of each test. Invasive tests detect genetic abnormalities other than aneuploidies, although the reason for consultation generally is an elevated risk for 21, T18 or T13, as estimated in CFTS. In addition, invasive testing can detect mosaic aneuploidy, with a mosaic percentage greater than 15–20 %. On another note, invasive prenatal testing entails a small risk of spontaneous abortion (0.1–0.2 %). The patient must also be aware that a high BMI can lead to no-calls. Regarding post-screening counseling, the patient should be informed that a high risk result must always be confirmed with an invasive test. Patients should also be aware that a low risk may be a FN or not exclude other genetic abnormalities not detected by NIPS.

## Implementation of NIPS in the Public Health System of Spain and the European Union

Neonatal screening protocols are changing as a result of the implementation of NIPS in many countries. The use of NIPS enables the detection of chromosomal aneuploidies and CNV. Hence, in some countries such as the Netherlands, CFTS is being replaced with ‘non invasive’ cfDNA testing. In Spain, NIPS is available in most of the autonomous communities, with different protocols being applied. In the public health system, NIPS is offered to the pregnant population with a high or moderate CFTS-estimated risk, both in singleton and multiple pregnancies (two fetuses maximum). When the risk is very high or in the presence of abnormalities in ultrasound, invasive testing is recommended to test for other types of genetic abnormalities.

In the EU, the level of implementation of NIPS is heterogeneous. This test has been included in prenatal screening in the public health system of 17 European countries. There is substantial variability in the inclusion criteria applied. In Belgium and the Netherlands, contingent screening has been replaced with universal cfDNA screening in the general pregnant population, regardless of their estimated risk. However, the first-trimester ultrasonography should not be eliminated in these cases, as it enables the detection of abnormalities in nuchal translucency, which are not only associated with T21, T13 and T18, but also with other disorders. Other countries such as Italy perform CFTS, and at a given level of risk, invasive testing or NIPS is recommended.

## Benefits and limitations of NIPS

In the clinic, NIPS facilitates early detection (≥10 gestational weeks) for chromosomal abnormalities, with a high sensitivity and specificity. Additionally, it provides crucial information for early decision making. NIPS is a robust test and, as mentioned above, it is integrated in the prenatal screening programs of most Autonomous Communities in Spain. This implementation has significantly reduced the number of invasive tests performed on women with high-risk pregnancies. Moreover, it results in a reduction of spontaneous abortions related to invasive testing (0.1–0.2 %), and a reduction of anxiety in future parents. TRIDENT is one of the largest studies performed to date. The first part (Trident-1) assessed the implementation of NIPS for aneuploidies following an initial screening [18]. The second part (Trident-2) was carried out to examine the role of NIPS as a universal prenatal screening test for the general pregnant population [20]. The results of the two parts of the study revealed that NIPS in women with a high risk for CFTS (1/200) is more accurate than traditional screening tests for T21, T18 and T13. The PPV of CFTS for the general pregnant population was 5.2, 4.1 and 1.4 for T21, T18 and T13, respectively vs. 93.5, 80 and 67 of NIPS for T21, T18 and T13, respectively. In addition, NIPS has a lower rate of FP, as compared to traditional screening tests [18]. On another note, Trident-2 revealed that NIPS is a universal screening test that does not require previous CFTS [20]. The study also demonstrated that NIPS is a robust screening test for detecting T21, T18 and T13 that can be integrated in national screening programs for fetal aneuploidies.

The role of genome-wide sequencing vs. targeted sequencing is still a matter of controversy, mainly due to the clinical relevance of incidental findings or detection of RAA. The Trident-2 follow-up results were recently analyzed to determine the clinical impact of screening according to laboratory results and perinatal events in the cases where an RAA was detected (0.36 % of pregnancies) [63]. Most fetal chromosomal abnormalities (22.1 % of RAAs) were pathogenic and associated with severe clinical phenotypes. As many as 53 % were placental mosaicisms, of which 8.5 % were associated with preeclampsia and 13.6 % with a low-birth-weight percentile.

One of the main limitations of NIPS is that it is a screening test, and the cases with a high risk of aneuploidy must be confirmed by an invasive test. Moreover, NIPS does not detect polyploidies (triploidies). The presence of low-level mosaicism or vanishing twins may lead to discordant results.

## Guidelines and recommendations

Scientific societies and organizations have published guidelines and recommendations on the implementation of NIPS for the detection of fetal aneuploidies in singleton and twin pregnancies. In 2023, the guidelines Noninvasive prenatal screening (NIPS) for fetal chromosome abnormalities in a general-risk population were publsihed by the American College of Medical Genetics and Genomics (ACMG), recommending cfDNA screening for the general pregnant population with singleton and twin gestations for fetal trisomies T21, T18, and T13, and RAA [64]. The International Society for Prenatal Diagnosis (ISPD) also recommends NIPS for trisomies T21, T18 and T13 in unselected singleton and twin gestations, or in pregnancies with a higher risk for fetal aneuploidy. Additionally, the ISPD considers NIPS accurate enough for detecting RAA. These societies concluded that NIPS is sensitive enough to be offered as a first line screening test for all pregnant women or as a contingent test [65].

The implementation of NIPS for the detection of RAA is controversial. Professional guidelines do not recommend its use due to the lack of robust evidence on its clinical validity. Although the PPV for (mosaic) RAAs is generally low, for some RAA it is comparable to that of T13 [66]. In addition, the effect of these RAAs on fetal development can be at least as severe as T21 and can be associated with adverse perinatal outcomes [67], 68].

## Conclusions

The implementation of NIPS in clinical practice has been a breakthrough in prenatal medicine. This test enables the early detection of fetal aneuploidies in maternal plasma samples, with a high sensitivity and specificity. In addition, NIPS significantly reduces the number of invasive procedures. NIPS is currently used in combined and universal screening for fetal aneuploidies. On another note, it is increasingly used for other purposes, including screening for pathologic point mutations responsible for diseases such as achondroplasia or cystic fibrosis. However, this test has some limitations in detecting other types of genetic abnormalities, especially when prenatal exome sequencing is performed using cfDNA samples. The future of non-invasive prenatal screening tests is promising to fetal medicine. Technological advances and emerging applications are expected to lead to the development of increasingly accurate prenatal diagnostic tools.
